# A critical review of the progress in prevention and treatment of radiation-induced skin damage

**DOI:** 10.3389/fonc.2024.1395778

**Published:** 2024-11-27

**Authors:** Li Kemin, Yin Rutie

**Affiliations:** ^1^ The Department of Obstetrics and Gynecology, West China Second University Hospital of Sichuan University, Chengdu, Sichuan, China; ^2^ Key Laboratory of Birth Defects and Related Diseases of Women and Children (Sichuan University), Ministry of Education, Chengdu, Sichuan, China

**Keywords:** radiation therapy, radiation-induced skin damage, review, progress, prevention, treatment

## Abstract

Radiation therapy was initially used in dermatology to treat various skin diseases, including acne vulgaris, keloids, plantar warts, tinea capitis and hirsutism. Although it is no longer used in the treatment of many of these diseases, radiation therapy still plays a crucial role in the treatment of keloids, skin cancer and solid organ malignancies. In the past 20 years, the widespread use of intensity-modulated radiation therapy has significantly increased in the management of tumor growth in multiple cancer sites and reduced the incidence of complications in normal organs. However, the occurrence and severity of radiation-induced organ complications still significantly affects the quality of life of patients and remains a research hotspot. Skin tissue is the largest area in the human body, serving as both a barrier and a defender. In patients undergoing radiation therapy, skin is often the first tissue that gets damaged. Especially, when the tumor involves the skin or is close to the skin (i.e., skin cancer, head and neck cancer, breast cancer, vulvar cancer), the treatment targets the superficial tissues, and may have inherent adverse effects on the skin. With the increasing incidence of cancer and the widespread use of radiation therapy in cancer treatment, the radiation-induced skin damage has become a serious problem. In this pursuit, the present study provides a review of the progress in the prevention and treatment of radiation-induced skin damage, thereby providing a reference for the prevention and treatment of radiation-induced skin damage.

## 1 Overview

Though radiation therapy exhibits a killing effect on the malignant tumor cells, it causes a serious damage to the normal tissue cells in the radiation area. In radiation therapy, the dose of radiation therapy, segmentation mode, type of radiation, treatment time, and radiation energy are all related to the severity of radiation injury. The severity of radiation-induced skin damage is also related to patient’s age, physical condition, skin type, treatment site, and other factors.

The main causes of radiation-induced skin damage (RIS) include nuclear radiation accidents, tumor radiotherapy, and occupational exposure. In tumor radiotherapy, the incidence of RIS is relatively high, with approximately 85% to 95% of tumor patients experiencing varying degrees of RIS. Nearly 60% of patients experience the RIS greater than or equal to CTCAE grade II, which greatly affects their quality of life leading to significant psychological and economic pressure. This may also lead to the discontinuation of treatment and significantly affecting the long-term prognosis in cancer patients (1–4). The occurrence and severity of RIS are related to many factors, including external and internal factors. External factors mainly include the total radiation therapy dose, dose fractionation mode, characteristics of the radiation, volume and surface area of the treatment area, etc. Internal factors mainly include the patient’s skin condition, nutritional status, age, race, various comorbidities including diabetes or connective tissue disease, etc. For example, the total radiation therapy dose is an important factor in the occurrence of RIS. The higher the radiation dose, the earlier the skin damage appears and the more severe it is.

The RIS exhibits important characteristics such as latency, timeliness, potential, progression, and persistence. Unlike ordinary burns and ulcers, radiation directly damages the skin and its deep tissue cells, leading to dryness, loss of elasticity, pigmentation, soft tissue fibrosis, capillary dilation, and radiation dermatitis in the irradiated area. In addition, it irreversibly damages the microvessels and endothelial cells in the skin tissue. As a result, the patient’s damaged skin does not heal for a long time and shows susceptibility to infection. Eventually, lesions develop into the fibrosis of skin tissue, and even cancer, thus significantly reducing the patient’s quality of life.

## 2 Classification and grading of RIS

According to the length of time between the end of treatment, RIS can be divided into acute radiation damage and chronic radiation damage. The skin damage that occurs during the radiation therapy or within one month after the treatment is defined as acute RIS. Skin damage that appears after more than one month after treatment is defined as chronic RIS (5, 6). Generally, RIS gradually worsens during treatment, with the severe period usually occurring in the last two weeks of radiation therapy and the first two weeks after treatment. Chronic RIS is usually associated with high-dose radiation and may cause thinning of the epidermis, dermal atrophy, edema, pigmentation, capillary dilation, ulcers or dermal necrosis, and fibrosis. Radiation-related skin fibrosis usually affects quality of life (5, 7, 8).

**Figure 1:**
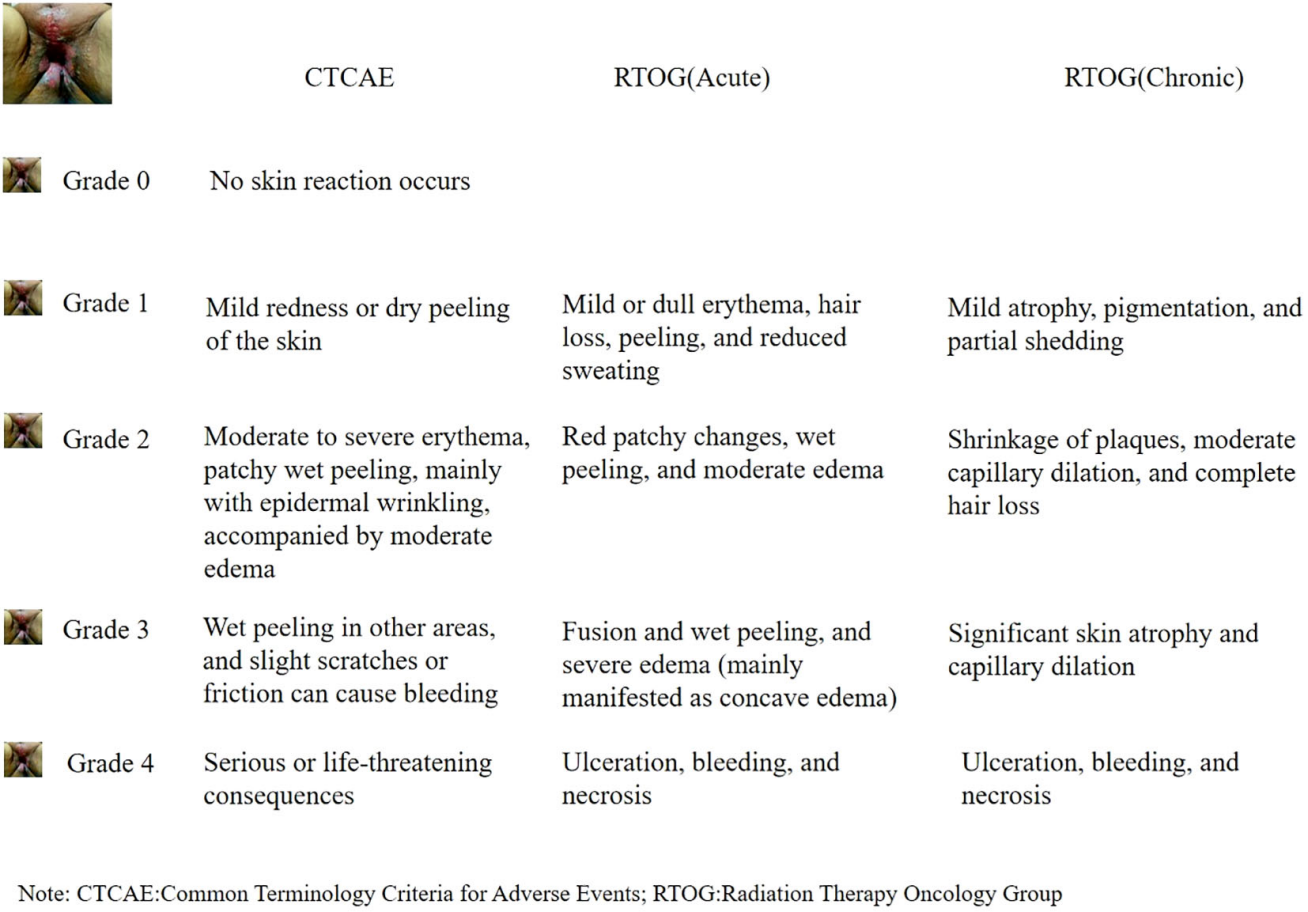
Classification and grading of RIS.

The grading tools for RIS in the clinical use are governed by the CTCAE standard (9) and the RTOG standard (10). It is shown in **Figure 1**. According to the CTCAE classification standard, it can be divided into five levels: 1) Grade 0: No skin reaction occurs; 2) Grade 1: Mild redness or dry peeling of the skin; 3) Grade 2: Moderate to severe erythema, patchy wet peeling, mainly with epidermal wrinkling, accompanied by moderate edema; 4) Grade 3: In addition to Grade 2 reactions, there is wet peeling in other areas, and slight scratches or friction can cause bleeding; 5) Grade 4: Serious or life-threatening consequences may occur, such as severe necrosis, large-scale dermal ulceration, and spontaneous bleeding at the affected area. According to the RTOG grading standard, Acute RIS can be divided into four levels: 1) Grade 1: Mild or dull erythema, hair loss, peeling, and reduced sweating; 2) Grade 2: Red patchy changes, wet peeling, and moderate edema; 3) Grade 3: In addition to Grade 1 and 2, there is fusion and wet peeling, and severe edema (mainly manifested as concave edema); 4) Grade 4: Ulceration, bleeding, and necrosis. Chronic RIS can be divided into four levels: 1) Mild atrophy, pigmentation, and partial shedding; 2) Shrinkage of plaques, moderate capillary dilation, and complete hair loss; 3) Significant skin atrophy and capillary dilation; 4) Ulceration, bleeding, and necrosis.

## 3 Prevention and treatment of RIS

Normally, the prevention and treatment of RIS should start with education and counseling before the radiation therapy. Currently, commonly used prevention and treatment methods in clinical practice include antioxidants, growth factor supplementation, antibacterial and anti-infection measures, as well as traditional Chinese medicine for the prevention or treatment. Nevertheless, the results are not ideal.

### 3.1 Prediction of RIS

There have been many studies on the assessment or prediction of the risk of acute RIS, but the results are divergent and there is currently a lack of unified standards. Recent research focuses on the predictive role of single nucleotide polymorphisms (SNPs) in RIS. In 2009, the Radiogenomics Consortium was established to collaborate on the establishment of large cohorts to study the association between the genetic markers (mainly SNPs) and adverse reactions to radiation therapy (11). A meta-analysis by Song Y.Z. et al. evaluated the association between the acute RIS and polymorphisms in the ERCC2 gene in breast cancer patients. The meta-analysis included 11 studies with a total of 2584 patients, and found a significant association between the ERCC2 rs13181 polymorphism and radiation toxicity (OR=0.71, 95% CI: 0.55-0.93, P=0.01) (12). Another meta-analysis by Su M. et al. evaluated the association between the SNPs in the ATM and TP53 genes and radiation toxicity, and included 20 studies. The overall analysis did not find a significant association between the ATM Asp1853Asn and TP53 Arg72Pro polymorphisms and the risk of adverse reactions to radiation therapy. The subgroup analysis by cancer type, tumor origin, and time of occurrence of side effects did not find a significant association between the ATM Asp1853Asn polymorphism and the risk of adverse reactions to radiation therapy. Stratified analysis by region showed that the TP53 Arg72Pro allele variation was associated with a reduced risk of adverse reactions to radiation therapy in Asian cancer patients (OR=0.71, 95% CI: 0.54-0.93, P=0.012) (13). Another meta-analysis by Zhao J. et al. evaluated the association between the SNPs in the XRCC1 gene and radiation therapy-related general tissue toxicity, and included 40 studies with a total of 6682 patients. The results showed that the rs25487 Arg399Gln polymorphism significantly increased the risk of acute radiation-induced side effects (OR=1.29, 95% CI: 1.10-1.52, P=0.002), especially acute mucositis (OR=1.91, 95% CI: 1.17-3.11, P=0.01) and acute gastrointestinal and genitourinary toxicity (OR=1.49, 95% CI: 1.04-2.11, P=0.03). In addition, the patients with the rs25487 Arg399Gln polymorphism were more likely to experience the radiation-induced side effects, when they had head and neck tumors (OR=1.46, 95% CI: 1.12-1.90, P=0.005) (14). A recent meta-analysis studied the prediction of acute radiation-induced dermatitis by SNPs, and included 16 cohorts with 4742 patients. The results showed that the most common SNPs were TGFβ1 rs1800469 (41%) and GSTA1 rs3957356 (36%), which were associated with skin radiation-induced adverse reactions. Seven genotypes that were associated with severe RIS, including PTTG1 rs3811999-CC; PTTG1 rs2961950-AA; MAD2L2 rs2294638-GG; MAT1A rs2282367-GG; GSTA1 rs3957356-CT; CD44 rs8193-CT; and SH3GL1 rs243336-GC. Five genotypes that were associated with lower RIS included PTTG1 rs2961952-GG; CD44 rs8193-CC; PTTG1 rs3811999-CT; MAT1A rs2282367-GA; and OGG1 rs2075747-AA) (15). Because the endpoints of studies related to RIS and SNPs are quite different, and different studies may choose different endpoints, such as acute mucositis, chronic mucositis, folliculitis, skin fibrosis, etc. There is still a lack of overlap in any SNP candidates across the cohort studies. Further research is needed in this area. Although the results of the existing studies still have a long way to go for the application in clinical practice, they fully demonstrate the predictive value of SNPs for severe acute RIS, which may provide opportunities for the development of personalized radiation therapy based on individual radiation sensitivity. This can also provide possibilities for reducing the risk and severity of RIS (16–18).

### 3.2 Education and nursing care for the prevention and treatment of RIS

Comprehensive skin protection for patients before, during, and after radiation therapy is an important task. This includes skin care, psychological care, and dietary care.

Skin care refers to the cleaning and care of a patient’s skin, which can effectively prevent wound infection, alleviate physical discomfort, and ensure the patient’s subsequent treatment (19–21). Patient education should promote personal and wound hygiene, promote comfort, prevent trauma to the damaged skin, and manage radiation dermatitis. Soft cotton clothing should be selected for patients to prevent excessive friction on the skin. The use of various irritating drugs or cosmetics must be strictly prohibited. In addition, the use of cold or heat sources (such as cold or hot compresses) should be prohibited. Hair on exposed parts of the body should not be shaved off during the course of the treatment (22–25).

The skin in the irradiated area should be kept dry and clean. Basic hygiene habits play an important role in the management of radiation-induced skin toxicity (19). Campbell I. R. et al. studied the protective effect of cleaning different radiation therapy areas on RIS. This was a randomized controlled study, with 99 patients receiving breast or chest wall adjuvant radiotherapy and randomly assigned three cleaning strategies: no cleaning, cleaning with water only, and cleaning with soap and water. The results showed that cleaning the skin in the radiation therapy area significantly reduced the incidence of acute RIS (20). Roy I. and others conducted a randomized controlled study and found that maintaining the cleanliness and hygiene of the radiation affected area significantly reduced the incidence and severity of RIS (21). Authoritative guidelines in the field of radiation therapy, such as ONS 2020 (22), SCoR 202 (23), and CCMB 2018 (24) also emphasize the importance of keeping the skin clean and dry in the irradiated area to alleviate the radiation-induced skin reactions. Further, they recommend the use of deodorant/antiperspirant + standard washing/skincare regimen or using standard washing/skincare regimen alone in individuals receiving trunk/chest radiotherapy.

Patients may experience varying degrees of fear/phobias before receiving radiation therapy. Healthcare professionals (including radiation oncologists, nurses, and technicians) should provide patients with the timely information on radiation therapy, various precautions, possible adverse reactions, and their corresponding measures. They should also inform the patients about the importance of timely skin care. Timely and effective communication can alleviate the fear and stress faced by the patients, and help them to perceive a more relaxed attitude towards the treatment. Song Y. et al. studied the impact of health education on radiation dermatitis in patients receiving head and neck radiation therapy. The researchers divided patients who met the inclusion criteria into two groups: 66 patients in the experimental group and 52 patients in the control group. The observation group received routine care, while the experimental group received team health education on the basis of the observation group. The results showed that health education significantly alleviated the discomfort and anxiety caused by radiation dermatitis, improved the patients’ quality of life and compliance with treatment, and improved the patients’ satisfaction of the treatment (25). Authoritative guidelines in the field of radiation therapy, such as ONS 2020 (22), SCoR 2024 (23), and CCMB 2018 (24) also emphasize the importance of health education before radiation therapy.

The skin is composed of several different layers, including the epidermis, dermis, white adipose tissue (WAT) in the dermis, and subcutaneous WAT. Mature adipocytes in the skin are mainly involved in several functional mechanisms, including lipid storage and release, adipokine secretion, glucose and lipid metabolism, and regeneration of hair follicles and fibroblasts after injury. Free fatty acids are relatively insoluble and potentially toxic, and can be transported to other cells through non-catalytic protein binding. A high-fat diet can increase the skin fat and resistance to the RIS (26). A high-fat diet may be an option during treatment, but whether it is beneficial for preventing or alleviating radiation damage needs further study.

### 3.3 Traditional Chinese medicine and herbal medicine

As one of the brilliant alternative system of medicine in China, traditional Chinese medicine (TCM) plays an indispensable role in the prosperity of China. It also has unique characteristics of diagnostic methods, systematic theoretical system, and significant therapeutic efficacy. Its rich historical materials and strong national cultural characteristics make it unique in the world of medicine. It has been recognized as a huge wealth for human health. TCM has been enduring for thousands of years and complements the modern medicine, showing strong vitality, which is also a major feature and advantage of the healthy development of Chinese medicine. In recent years, TCM has been widely used in the prevention and treatment of RIS, and has shown good therapeutic effects. Ding T. et al. (27) used meta-analysis to evaluate the clinical efficacy of TCM external treatment in acute radiation dermatitis. The study included 17 clinical studies and 1,453 patients. The results showed that compared with the Western medicine group, TCM external treatment significantly improved the clinical efficacy of acute radiation dermatitis, RR=1.27, 95% CI (1.17, 1.39), P<0.01. Further, the prophylactic medication significantly improved the patient symptoms, and TCM external treatment was more effective than the Western medicine group in preventing the severity of RIS, RR=0.25, 95% CI (0.16, 0.38), P<0.01. TCM external treatment effectively shortened the clinical cure time [MD=-4.28, 95% CI (-6.18, -2.38), P<0.01] and improved the quality of life of the patients to a certain extent [MD=3.17, 95% CI (0.15, 7.27), P=0.04]. Kalekhan F. et al. (28) reviewed the effectiveness of the traditional Chinese medicine in preventing the RIS and systematically analyzed the preventive and therapeutic effects of drugs such as Coix seed bran extract, Aloe vera, epigallocatechin-3-gallate, honey, Chinese yam, chamomile, olive oil, and compound ointment on RIS. The results showed that these traditional Chinese medicines significantly reduced the risk and severity of the radiation dermatitis, which is worthy of a clinical promotion, but the relevant mechanisms need further research. Currently, commonly used TCM drugs in clinical practice mainly include Kangfu Xin liquid, purple gromwell oil, Aloe vera, and dressing-type Chinese medicines (29–32).

### 3.4 Western medicine and drugs

Western medicine has always played a leading role in the prevention and treatment of radiation damage. Commonly used Western medicines in clinical practice include antioxidants, epidermal growth factors, hormone drugs, etc.

Vitamins act as antioxidants and mitigate the free radicals. It can maintain enzyme activity in patients, enhance tissue function of biological membranes and mitochondria, clear free radicals in the body, and effectively helps in the treatment of the RIS. Moreover, it can participate in normal metabolism of the human body and help the body to repair the damaged skin epithelial cells. Pantothenic acid (vitamin B5) is essential in the metabolism and maintaining skin integrity. Pantothenic acid deficiency can cause dermatitis, while excess pantothenic acid can promote epithelial regeneration. Vitamin drugs as antioxidant drugs have been widely used in clinical prevention and treatment of radiation damage.

Hyaluronic acid is a commonly used endogenous drug, which is a carbohydrate polymer distributed throughout the connective tissue. It is an important component of the dermis. Primavera G (33) conducted a randomized controlled study to evaluate the preventive and therapeutic effects of hyaluronic acid on radiation dermatitis. A study found that sodium hyaluronate offered a certain preventive and therapeutic effect on the RIS, and also exhibited a certain improvement effect on its clinical manifestations.

Epidermal growth factor (EGF) plays a critical role in promoting the proliferation of human fibroblasts, epidermal stem cells, and epidermal cells. Studies have shown that EGF released by platelets, macrophages, and fibroblasts increases in acute wounds and helps healing by promoting the epithelial regeneration (34). Kang H. C. and others conducted a multicenter clinical study on the safety and efficacy of EGF-based cream in preventing RIS (35). The results showed that prophylactic use of EGF-based cream could effectively prevent the radiation dermatitis, and exhibited a tolerable toxicity.

Triethanolamine cream is a compound preparation with good moisturizing properties. Applying it to the damaged skin of patients can clean the area and effectively reduce the skin dryness, inflammation and edema, promote microcirculation in the body, enhance skin tolerance, and accelerate wound healing. Studies have shown that prophylactic use of triethanolamine cream can effectively prevent the RIS, delay the onset of radiation dermatitis, and accelerate the recovery of skin lesions, thereby relieving patient suffering. It is worth promoting in clinical practice (36–38).

Corticosteroids are a type of steroidal drugs that exhibit strong anti-inflammatory effects. They are commonly used to treat radiation-induced dermatitis, since they can inhibit the proliferation of radiation-induced cytokines (3). Topical glucocorticoids have significant therapeutic effects on RIS by not only reducing its incidence, but also alleviating its severity and improving the quality of life. Ho A. Y. et al. (39) conducted a double-blind randomized trial to evaluate the efficacy of 0.1% mometasone furoate (MF) and hydrocortisone cream in preventing moderate to severe acute radiation dermatitis (ARD) in breast cancer patients receiving treatment. The results showed that the wet desquamation rate of the entire cohort was 54.8%, and the incidence rate in the MF group was significantly lower than that in the control group (43.8% vs. 66.7%; P=0.012). The incidence of severe skin toxicity in the MF group was lower (P=0.036), and the time to develop grade 3 dermatitis was longer (46 days vs. 35.5 days, P ≤ 0.001). Haruna F. et al. (40) systematically evaluated the efficacy of topical corticosteroids in treating acute radiation dermatitis in female breast cancer patients. The results showed that in 10 studies involving 919 patients, local corticosteroids significantly reduced the incidence of wet desquamation (OR=0.29, 95%CI (0.19-0.45), p<0.0001) and decreased the mean radiation dermatitis score (SMD: -0.47, 95%CI: -0.61- -0.33, p<0.00001).

Currently, there are about ten Western medicines used in the clinical practice, including interleukins, ketocyclazocine, statins, silver sulfamethoxazole, and platelet-rich plasma. However, there is a limited clinical research data and the clinical efficacy still needs a further study.

### 3.5 Mesenchymal stem cell therapy

The use of allogeneic stem cells in RIS treatment is one of the most important research topics in regenerative medicine for radiation damage. The presence of stem cells can increase the number of functional cells in the affected area of the skin. Adipose-derived mesenchymal stem cells can help to heal the injured tissue by secreting growth factors in the tissue that provides appropriate environmental conditions. The presence, migration, proliferation, and differentiation of mesenchymal stem cells in the treated tissue may provide a promising treatment for alleviating radiation-induced skin tissue damage (41–44). Huayllani M. T. et al. (42) systematically evaluated the application value of mesenchymal stem cell therapy in RIS, including 8 studies that met the criteria. The results showed that this treatment method could significantly improve the wound healing. Mesenchymal stem cell therapy is currently limited to animal models and lacks clinical data.

### 3.6 Tetrahydrobiopterin

Tetrahydrobiopterin (BH4) is a key cofactor of nitric oxide synthase (NOS), also known as sepiapterin. Under normal physiological conditions, the activity of guanosine triphosphate cyclohydrolase 1 (GCH1) decreases during the BH4 biosynthesis process, leading to a decrease in BH4 production, NOS uncoupling, increase in reactive oxygen species (ROS), and thus causing or exacerbating radiation damage. It has been reported that activation of GCH1 increased the BH4 levels and NO production in irradiated epidermal cells, eliminated ROS, reduced DNA damage, and reduced cell aging and apoptosis. Our previous research found that the application of BH4 in the skin alleviated the RIS, promoted damage repair, reduced skin tissue damage after radiation, and maintained normal skin physiological function (45–47). Clinical data on tetrahydrobiopterin is still lacking, and current research is mainly focused on the basic research. There are registered clinical trials studying its prevention and treatment of RIS, and the results may guide its clinical application.

### 3.7 Physical therapy

The physical treatment for RIS mainly refers to hyperbaric oxygen therapy, which is commonly used for patients with persistent late-stage RIS (48, 49). It involves the use of 100% oxygen under pressure higher than atmospheric pressure. Oxygen therapy for skin lesions can effectively increase the oxygen supply to the skin lesions, reduce inflammatory exudation of the wound, and accelerate the wound drying and healing.

### 3.8 Management of adverse drug reactions

Virtually all drugs have the potential to cause adverse reactions. Drugs used in RIS that more commonly cause adverse reactions include Western medicine and drugs, and Traditional Chinese Medicine and Herbal Medicine. In most cases, drugs used to treat or prevent RIS are safe and rarely cause serious adverse reactions. Most adverse reactions are mild, such as itching, eczema, mild pain, redness, swelling, etc. Patients and their families should be counseled about the possibility of a drug reaction and the signs and symptoms of one. Patients should be told to report any signs and symptoms of a drug reaction, especially after they have left the clinic. The management recommendations depend on the severity of the reaction and the type of drug that caused the reaction. Typically, the drug should be stopped for patients having a reaction. The one exception to this rule is that mild reactions usually resolve after stopping the drug. Whereas H1 blocker antihistamine such as diphenhydramine or hydroxyzine is recommended for managing drug reactions. Corticosteroids are also generally reserved for mild reactions, if symptoms do not quickly resolve after administering an H1 blocker.

## 4 Summary

The indirect mechanism of ionizing radiation can cause radioactive damage to the skin. Most malignant tumor patients experience varying degrees of RIS during and after the radiation therapy, which may significantly affect the treatment process and quality of life of the patients. At the same time, RIS can cause mental and physical problems, thereby affecting the patient treatment and survival. Hence, RIS has become a serious clinical problem. The prevention and treatment of RIS has gradually attracted attention, and various intervention measures that can significantly reduce its incidence and severity are becoming an important auxiliary method in tumor treatment. Traditional Chinese medicine plays an important role in the prevention and treatment of the RIS, and various Western medicine treatment methods have also achieved significant preventive and therapeutic effects. With the continuous development of the scientific and technological progress and clinical research, new treatment methods are constantly emerging, which are expected to bring more good news to patients with RIS.
